# Does neuregulin-1 play a role in Type A behavior? The cardiovascular risk in young Finns study

**DOI:** 10.1186/1744-9081-4-40

**Published:** 2008-09-17

**Authors:** Helena M Service, Mirka Hintsanen, Taina Hintsa, Terho Lehtimäki, Olli T Raitakari, Jorma S Viikari, Liisa Keltikangas-Järvinen

**Affiliations:** 1 Department of Psychology, University of Helsinki, Finland; 2 Laboratory of Atherosclerosis Genetics, Department of Clinical Chemistry, Centre for Laboratory Medicine, Tampere University Hospital, Finland; 3 Medical School, University of Tampere, Finland; 4 Department of Clinical Physiology, University of Turku, Finland; 5 Department of Medicine, University of Turku, Finland

## Abstract

**Background:**

Neuregulin-1 proteins are related to physiological correlates of Type A in terms of cardiac reactivity. Furthermore, neuregulin-1 gene (NRG1) may play a role in cardiovascular disease such as atherosclerosis and coronary heart disease i.e. the suggested "outcomes" of Type A behavior. Therefore, NRG1 is hypothesized to be associated with Type A behavior.

**Methods:**

The study examined whether Type A behavior pattern is associated with the single nucleotide polymorphism (SNP) SNP8NRG221533 of the NRG1. The subjects were 631 men and women participating in the population-based Cardiovascular Risk in Young Finns study in 1992 and 2001. Type A was self-assessed with the Framingham Type A Scale and reassessed nine years later.

**Results:**

Type A was associated with NRG1 genotype. Carriers of genotype CC scored lower on Type A compared to the others.

**Conclusion:**

Our study has pinpointed a SNP in NRG1 that predicts Type A behavior. As previous evidence suggests an association for NRG1 with beta-adrenergic stimulation, its role underlying Type A is discussed.

## Background

Type A behavior, originally described as a behavioral pattern comprising impatience, hard driving and a sense of hurry [1], was considered in the 60s and 70s as a major behavioral risk factor for coronary heart disease (CHD). It was believed to have a similar effect on cardiovascular risk as the more traditional risk factors, such as elevated systolic blood pressure, serum cholesterol, and smoking [2].

In the 1980's, however, most studies failed to confirm an association between Type A behavior and CHD [3]. In 1999, the review by Hemingway and Marmot [4] concluded that a contribution of Type A behavior in pathogenesis of CHD has not been scholarly proved, while in 2000's, new evidence on an association between Type A and atherosclerosis has been, again, elicited [5].

Vagueness of the Type A concept may at least partly, explain these conflicting findings. There is a disagreement whether Type A mainly refers to emotions, attitudes, behavioral styles, or innate dispositions, and what is the contribution of those dimensions to the final concept. If Type A behavior, or any dimension of it, could be anchored to genetic background, this might establish its content. More important, identifying functional genes related to Type A behavior might increase our knowledge about mechanisms through which Type A could be associated with health outcomes. This study was taken with these purposes. As far as we know, there are no previous studies that have associated Type A behavior with molecular genetics. For two reasons, neuregulin-1 (NRG1) might belong to candidate genes to start with. First, neuregulin-1 proteins are related to physiological correlates of Type A in terms of cardiac reactivity. They seem to reduce excessive beta-adrenergic stimulation, and help to produce counterbalancing parasympathetic activity [6,7], and Type A behavior has been linked to the sensitivity of the beta-adrenergic system [8,9] and sympathetic versus parasympathetic balance [10]. Second, NRG1 may play a role in cardiovascular disease such as coronary heart disease and atherosclerosis [11] i.e. the suggested "outcomes" of Type A behavior. The present study examines an association of the single nucleotide polymorphism SNP8NRG221533 in the NRG1 gene with Type A behavior.

## Methods

### Participants

The subjects were derived from the ongoing prospective population based study, called Cardiovascular Risk in Young Finns. The Young Finns study has followed a random sample of 3596 healthy Finnish children and adolescents (3–18 years old at baseline) since 1980 [12].

Genetic data was acquired from a sub-sample of 1600 subjects (missing n = 62). Psychological data on adult Type A behavior was obtained first in 1992, and again in 2001 when participants were 15–30 and 24–39 years old, respectively.

Participants under the age of twenty at the first Type A assessment were excluded, as questions concerning job-related Type A behavior were not age-appropriate for them. After this exclusion, psychological data was received from 631 genotyped participants who had complete Type A measures from both follow-ups. Of them 276 (43.7%) were men and 355 (56.3%) women. Participants gave written informed consent, and the study was approved by local ethics committees.

### NRG1 genotyping

Genomic DNA was extracted from peripheral blood leukocytes using a commercially available kit (Qiagen Inc, Hilden, Germany) and DNA samples were then genotyped by employing the 5'exonuclease assay [13]. For the PCR, primers and allele-specific fluorogenic probes with conjugated minor groove binder groups were synthesized in conjugation with Applied Biosystems (Foster City, CA, USA) using the sequence (SNP8NRG221533) found on the deCODE Genetics Web site [14] and GenBank (accession number AF491780). The PCR reaction mixture consisted of genomic DNA, 1 × Universal PCR Master Mix, 900 nM of each primer and 200 nM of each probe. Amplification was performed using the TaqMan Universal Thermal Cycling Protocol. After PCR, end-point fluorescence intensity was measured by the ABI Prism 7900HT Sequence Detection System (Applied Biosystems, Foster City, CA, USA) and allelic discrimination performed. All genotyping was performed blinded to participant outcome. Negative controls (water) and random duplicates were used as quality control.

### Type A behavior assessment

Type A behavior was self-assessed with the Framingham Type A Scale [15]. The scale is composed of two subscales: trait-related Type A behavior and job-related Type A behavior. Trait-related statements are mainly related to general feelings such as a sense of competitiveness and hurry e.g. (1) being hard-driving and competitive and (2) being bossy and dominating. The job-related questions are directly related to work e.g. (1) work often stretching an individual to the very limits of their energy and work capacity and (2) work often staying with an individual so that they were thinking about work after working hours. There were five items on trait-related scale and four items on job-related scale. The mean score of the sub-scales can be used as a measure of overall Type A. A high score indicated a high amount of Type A behavior. The Cronbach's alphas ranged from 0.6 to 0.7 for trait-related Type A and for the overall Type A. Alpha of job-related scale was 0.5.

### Statistical analysis

Univariate ANOVAs were used to determine differences between genotypes in overall Type A behavior and its sub-scales for measurements in 1992 and 2001 and for the mean values of the two assessments. Effect sizes were calculated with partial eta squared (η^2^). Genotypes TT and TC were combined as mean values of Type A in these genotypes were almost identical (see Table 1). Analyses were adjusted for age and gender. Data analysis was carried out using SPSS/Win (Version 15.0) software. The χ^2^-test was used for calculation of the Hardy-Weinberg equilibrium.

**Table 1:** Characteristics of Type A Measurements in NRG1 Genotypes.

	Trait-related Type A^1^	Job-related Type A^1^	Total Type A^1^
			
TT (n = 214)	2.50 ± 0.50	1.46 ± 0.24	1.98 ± 0.30
TC (n = 311)	2.50 ± 0.47	1.45 ± 0.24	1.98 ± 0.29
CC (n = 106)	2.37 ± 0.46	1.42 ± 0.25	1.90 ± 0.29

Values represent means ± 1 standard deviations.

TT, TC, and CC represent genotypes of neuregulin-1 gene.

^1^Type A variables present the means of two measurements (1992 and 2001).

## Results

### Sample representativeness

The group with data from all used variables was compared to the excluded cases with t- and χ^2^-tests to assess representativeness. No differences were found in NRG1 genotype frequencies or Type A behavior (tested using the mean values of the two measurements). However, women were somewhat overrepresented in the data (56.3% of included and 49.8% of the excluded participants were women, p = .003). Included participants were naturally also older than excluded participants, because of our age criterion.

### Sample characteristics

The percentages of subjects carrying genotypes TT, CT and CC in the whole genotyped sub-sample (n = 1600, missing n = 62), were 33.7%, 49.7% and 16.6%, respectively. The genotype frequencies followed Hardy-Weinberg equilibrium (p = .83). Table 1 presents means and standard deviations of trait-related Type A, job-related Type A, and Total Type A across the NRG1 genotypes.

### NRG1 genotype and Type A behavior

NRG1 genotype was significantly associated with trait-related Type A behavior (p = .01, η^2 ^= .010) and overall Type A behavior (p = .02, η^2 ^= .009) on first assessment in 1992, genotype CC carriers having lower Type A than others. These associations were replicated on second assessment in 2001 (trait-related Type A: p = .05, η^2 ^= .006; overall Type A: p = .04, η^2 ^= .007) and when Type A was calculated as the mean of the two assessments in1992 and 2001 (trait-related Type A: p = .01, η^2 ^= .010; overall Type A: p = .01, η^2 ^= .010). Again genotype CC carriers showed lower Type A compared to others. Job related Type A was not associated with NRG1 genotype on either assessment or in mean of the two assessments (p > .24 in all measurements).

## Discussion

Our findings showed that when compared with other genotypes, individuals carrying the genotype CC of NRG1 had lower Type A behavior. This was true with total Type A and its trait-related subscale.

The trait-related subscale differentiated the genotypes whereas the job-related subscale did not. This would be in line with an assumption that trait-related Type A behavior is more innate and job-related behavior more circumstantial. It should be noted that calculating the total Type A behavior in the way used, also emphasizes the portion of trait-related Type A compared to job-related Type A behavior.

As the etiology of Type A is still unclear, an association between NRG1 and Type A behavior might provide hints of the physiological basis of this personality type and imply a mechanism through which Type A behavior may have its effect on health. NRG1 plays a part in the survival, growth and repair of adult cardiomyocytes as a response to increased workload [16]. It is also crucial in the development of the autonomic nervous system [16] and the heart [17]. Therefore some autonomic or cardiostructural function of NRG1 could be related to Type A. One intriguing possibility for explaining the association is the adrenergic system. Type A behavior has been associated with increased sympathetic balance compared to parasympathetic tone [10,18]. It has been linked, in particular, to the beta-adrenergic system in men, as it has been associated with greater B2 adrenergic receptor density [8], and in a more recent study to greater sensitivity to B1 and B2 receptor agonist isoproterenol administered by bolus injection [9]. In Type A men, isoproterenol has a stronger effect on the T wave of the cardioelectrogram, and parasympathetic antagonism of beta-adrenergic stimulation also appears to be less effective [10].

Neuregulin proteins, on their part, appear to have the ability to control excessive beta-adrenergic activation. This is, incidentally, likely to be a key factor in neuregulin's protective role in heart failure [6,7]. In animal studies, neuregulin-1 has been shown to induce counterbalancing parasympathetic activity [7] and to directly reduce contractibility in heart muscle cells [6]. Thus, adrenergic responses might play a role in the etiology of Type A and would be an interesting focus of further molecular research.

It has primarily been through responses to stressors that Type A has been thought to be pathogenic, even when considered as risk factor for atherosclerosis [19] and not just a trigger for coronary events. If Type A behavior is, indeed, an actual risk factor for atherosclerosis, neuregulin could be involved in additional ways. As mentioned above, neuregulin has a protective role in heart failure. In rat cardiomyocytes, neuregulin-1 activates endothelial nitric oxide synthase [6], and there is evidence that the nitric oxide synthase has a role in both heart failure and atherosclerosis [20,21]. Although neuregulin proteins are beneficial in heart failure, they may, in fact, play a pathological role in atherosclerosis.

Neuregulin has been found to be overexpressed in coronary atherosclerotic lesions in the intima of human blood vessels, primarily in macrophages [11]. Type A, in turn, has been associated with coronary atherosclerosis in Sparagon's study with male subjects [22] and also with markers of arteriosclerosis [23]. The different NRG1 genotypes might, therefore, mark variation in both susceptibility to Type A and the development of atherosclerosis. This hypothesis must, however, be considered with caution, as the link between different NRG1 forms and atherosclerotic factors has not yet been thoroughly investigated.

## Limitations

Cronbach's alpha reliabilities of Type A measures were moderate in size varying mostly between 0.6 – 0.7. Previously reported reliabilities for overall Type A correspond to these values [24]. In the current study job-related Type A had a slightly lower reliability (0.5). Reliabilities for Type A subscales are seldom reported but it is likely that their reliabilities are in general somewhat lower than that of the overall Type A scale's, as subscales have fewer items, which in turn, has a diminishing effect on Cronbach's alpha values.

It is important to note that the association between NRG1 genotype and Type A behavior was found in Framingham Type A. It is known that different Type A measures do not correlate very strongly as they emphasize different aspects of the multidimensional concept. The current results suggest an association for NRG1 with Framingham Type A but not necessarily with Type A behavior defined by other measures.

## Conclusion

To our knowledge, our study is the first to examine molecular genetics and Type A. A genetic component has previously been suggested on the basis of quantitative genetics [25]. A follow-up of nine years gives credit to the suggestion that NRG1 might belong to a genetic basis of a stable Type A personality.

## Abbreviations

SNP: single nucleotide polymorphism; CHD: coronary heart disease; NRG1: neuregulin-1; DNA: deoxyribonucleic acid; PCR: polymerase chain reaction

## Competing interests

The authors declare that they have no competing interests.

## Authors' contributions

HMS participated in the design of the manuscript, performed the statistical analyses and drafted the manuscript. MH participated in the design of the manuscript, performed the statistical analyses and drafted the manuscript. TH helped to perform the statistical analyses and helped to draft the manuscript. TL was responsible for the molecular genetic studies, and helped to draft the manuscript. OTR participated in collection of data, and helped to draft the manuscript. JSV participated in the collection of data, helped to draft the manuscript. LKJ conceived of the study and participated in its design and coordination and collection of data, and drafted the manuscript. All authors read and approved the final manuscript.
